# A quantitative analysis of cohesin decay in mitotic fidelity

**DOI:** 10.1083/jcb.201801111

**Published:** 2018-10-01

**Authors:** Sara Carvalhal, Alexandra Tavares, Mariana B. Santos, Mihailo Mirkovic, Raquel A. Oliveira

**Affiliations:** Instituto Gulbenkian de Ciência, Oeiras, Portugal

## Abstract

Sister chromatid cohesion mediated by cohesin is essential for mitotic fidelity. It counteracts spindle forces to prevent premature chromatid individualization and random genome segregation. However, it is unclear what effects a partial decline of cohesin may have on chromosome organization. In this study, we provide a quantitative analysis of cohesin decay by inducing acute removal of defined amounts of cohesin from metaphase-arrested chromosomes. We demonstrate that sister chromatid cohesion is very resistant to cohesin loss as chromatid disjunction is only observed when chromosomes lose >80% of bound cohesin. Removal close to this threshold leads to chromosomes that are still cohered but display compromised chromosome alignment and unstable spindle attachments. Partial cohesin decay leads to increased duration of mitosis and susceptibility to errors in chromosome segregation. We propose that high cohesin density ensures centromeric chromatin rigidity necessary to maintain a force balance with the mitotic spindle. Partial cohesin loss may lead to chromosome segregation errors even when sister chromatid cohesion is fulfilled.

## Abstract (precis)

Sister chromatid cohesion is a prerequisite for faithful mitosis. Through artificial removal of precise amounts of cohesin from metaphase chromosomes, Carvalhal et al. show that sister chromatid cohesion is highly resistant to cohesin loss. Nevertheless, partial cohesin decay compromises mitotic fidelity by impairing chromosome attachments.

## Graphical Abstract

**Figure:**
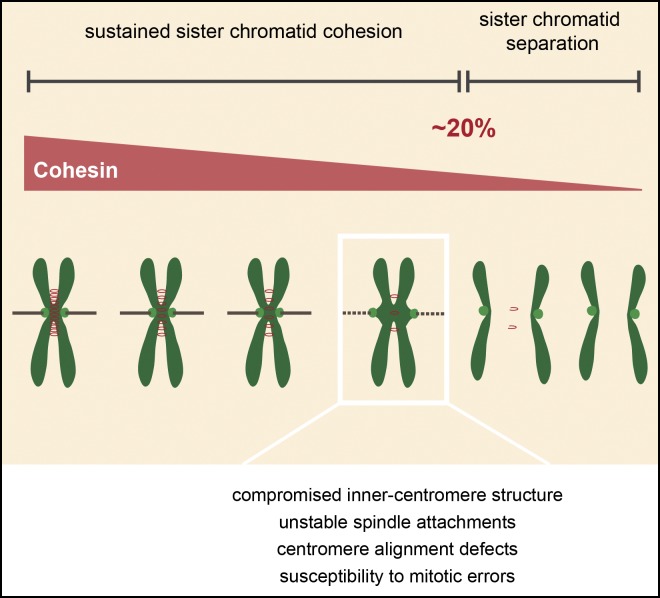

## Introduction

Maintenance of sister chromatid cohesion from the time of DNA replication until the late stages of mitosis is required for faithful chromosome segregation. This process is mediated by cohesin, a ring-like complex composed of two SMC proteins (Smc1 and Smc3) bridged by Rad21/Scc1 (Guacci et al., 1997; Michaelis et al., 1997). Cohesin topologically entraps sister DNA molecules inside its ring (Haering et al., 2008). Upon entry into mitosis, most of cohesins along chromosome arms are removed in a cleavage-independent manner, whereas centromeric complexes are retained (Losada et al., 1998; Waizenegger et al., 2000; Warren et al., 2000). At anaphase onset, cleavage of Scc1/Rad21 by Separase opens cohesin rings and releases chromatids, allowing spindle forces to move them apart and conduct poleward chromosome motion (Uhlmann et al., 1999, 2000; Oliveira and Nasmyth, 2010).

During metaphase, cohesins present the sole force counteracting spindle microtubule-pulling forces, and artificial removal of this complex is sufficient to trigger sister chromatid disjunction (Uhlmann et al., 2000; Oliveira et al., 2010). This is remarkable as the mitotic spindle exerts forces of ∼700 pN on chromosomes (Nicklas, 1983; Ye et al., 2016). Indeed, sister chromatid cohesion is known to surrender to spindle forces in cells arrested in mitosis for long periods, leading to sister chromatid separation, also known as cohesion fatigue (Daum et al., 2011). Regulation of cohesion establishment/maintenance is orchestrated by numerous factors that prevent premature sister chromatid separation (PSCS) and consequent aneuploidy (Mirkovic and Oliveira, 2017).

Several studies report decreased cohesin levels in some potential pathological conditions such as cancer (Losada, 2014; De Koninck and Losada, 2016) and age-related female infertility (Webster and Schuh, 2016). However, how much cohesin levels impact on chromosome cohesion in metazoans has never been approached in a quantitative manner. Pioneering research in budding yeast reveal that strains expressing solely 13% Rad21/Mcd1 do not display evident cohesion defects, whereas other cohesin functions are affected (Heidinger-Pauli et al., 2010). However, levels of chromosome-bound cohesin did not linearly correlate with total protein amounts in those strains, suggesting that compensatory mechanisms may enhance cohesin loading and/or stability. Moreover, yeast cells lack a prophase pathway, and so how these findings translate to metazoan organisms remains unknown.

To bypass caveats of potential adaptive mechanisms, we developed a system to acutely remove well-defined levels of cohesin from preestablished metaphase chromosomes, providing a quantitative view on immediate consequences of cohesin loss in metazoan chromosomal architecture and the implications on their faithful segregation.

## Results and discussion

### A system to acutely remove variable amounts of cohesin complexes from metaphase chromosomes

**Figure 1.**
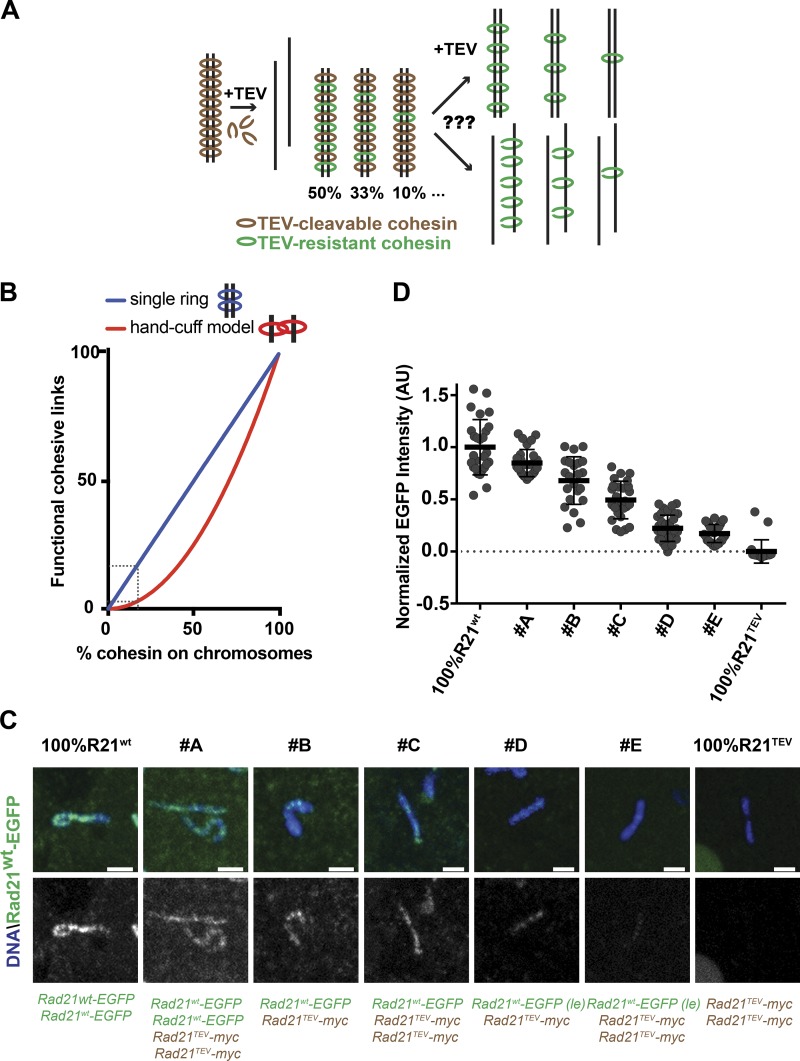
**A system to acutely remove variable cohesin amounts from metaphase chromosomes. (A)** Experimental approach used to titrate the amount of TEV-resistant cohesin complexes (green circles) upon TEV-induced removal of TEV-cleavable cohesin fraction (brown circles). Variable ratios of these two cohesin versions allow testing of how different cohesin levels sustain chromatid cohesion. **(B)** Probabilistic models for functional cohesive links (y) relative to cohesin left on chromosomes (x) based on possible models for cohesion. Single ring model: y = x. Handcuff model: y = (x/100)^2^, assuming random and independent distribution of TEV-resistant/TEV-cleavable rings. Dashed lines correspond with the cohesin level threshold identified in this study. **(C)** Representative chromosome spreads of Rad21^WT^-EGFP (green) and DNA (Hoechst) from the established strains. Bars, 2 µm. **(D)** Relative mean fluorescence intensity of Rad21^WT^-EGFP within chromosomal area (Hoechst staining) measured in different strains and normalized for 100% Rad21^WT^. *n* = 27, 28, 22, 26, 36, 29, and 21 individual chromosomes from at least three independent embryo spreads. Mean ± SD; see also Fig. S1.

To address how specific quantities of cohesin complexes sustain sister chromatid cohesion, we developed a layout to remove defined amounts of cohesin from metaphase chromosomes. We used a system that enables fast cohesin inactivation by the tobacco etch virus (TEV) protease in *Drosophila melanogaster* strains carrying a modified version of Rad21 containing TEV cleavage sites (Pauli et al., 2008). TEV protease injection into *Drosophila* early embryos enables acute cohesin loss, triggering sister chromatid separation within minutes (Oliveira et al., 2010). Specific ratios of TEV-cleavable and TEV-resistant forms of Rad21 allowed us to titrate the amount of Rad21-containing complexes resistant to TEV protease (Fig. 1 A). Using the assumption that a single ring embraces two sister chromatids (Haering et al., 2008), removal of a precise percentage of cohesin molecules should imply a direct loss of an equal amount of cohesive links (Fig. 1 B). Models that predict two rings (e.g., the handcuff model; Zhang et al., 2008) would result in a more pronounced loss of functional cohesion relative to cohesin levels. According to this model, functional cohesive links will only be maintained in connections built from two TEV-resistant Rad21 molecules whose probability of occurrence decreases in a nonlinear manner upon reduction of Rad21^WT^ (Fig. 1 B).

*Drosophila* strains carrying different combinations of ectopic constructs expressing both TEV-sensitive and TEV-resistant Rad21 molecules were produced (referred as strains A–E; see Fig. S1 A for details). We used transgenes that express Rad21^WT^ or Rad21^TEV^ at levels similar to endogenous WT Rad21 (Fig. S1 B). Additionally, we took advantage of a strain expressing lower levels of Rad21^WT^, referred as low expression–Rad21^WT^, possibly as a result of transgene chromosomal positioning (Fig. S1 B).

Combinations of these transgenes resulted in variable levels of total Rad21 available with different ratios of TEV-sensitive and TEV-resistant Rad21 complexes (Fig. S1, A, C, and D). To quantify cohesin levels that would remain on mitotic chromosomes, we performed imaging analysis on native chromosome spreads from metaphase-arrested and staged embryos to measure the mean pixel intensity of TEV-resistant complexes (labeled by Rad21^WT^-EGFP) within the chromosomal area (defined by Hoechst). The results indicate a gradual decline of Rad21^WT^-EGFP across the established strains (Fig. 1, C and D) leading to a homogeneous decrease of chromosome-bound Rad21^WT^-EGFP along the chromosome length (Fig. S1 E). The values obtained correlate with predicted amounts based on genetic background, although total quantity of Rad21 available also impacts on chromosome-bound cohesin levels (see Fig. S1 A). Given the high efficiency of TEV-mediated Rad21^TEV^-myc cleavage (Fig. S1 F), this system enables acute inactivation of Rad21^TEV^, whereas variable amounts of Rad21^WT^ remain intact (see example in Fig. S1, G and H).

### Sister chromatid cohesion is highly resistant to cohesin loss

**Figure 2.**
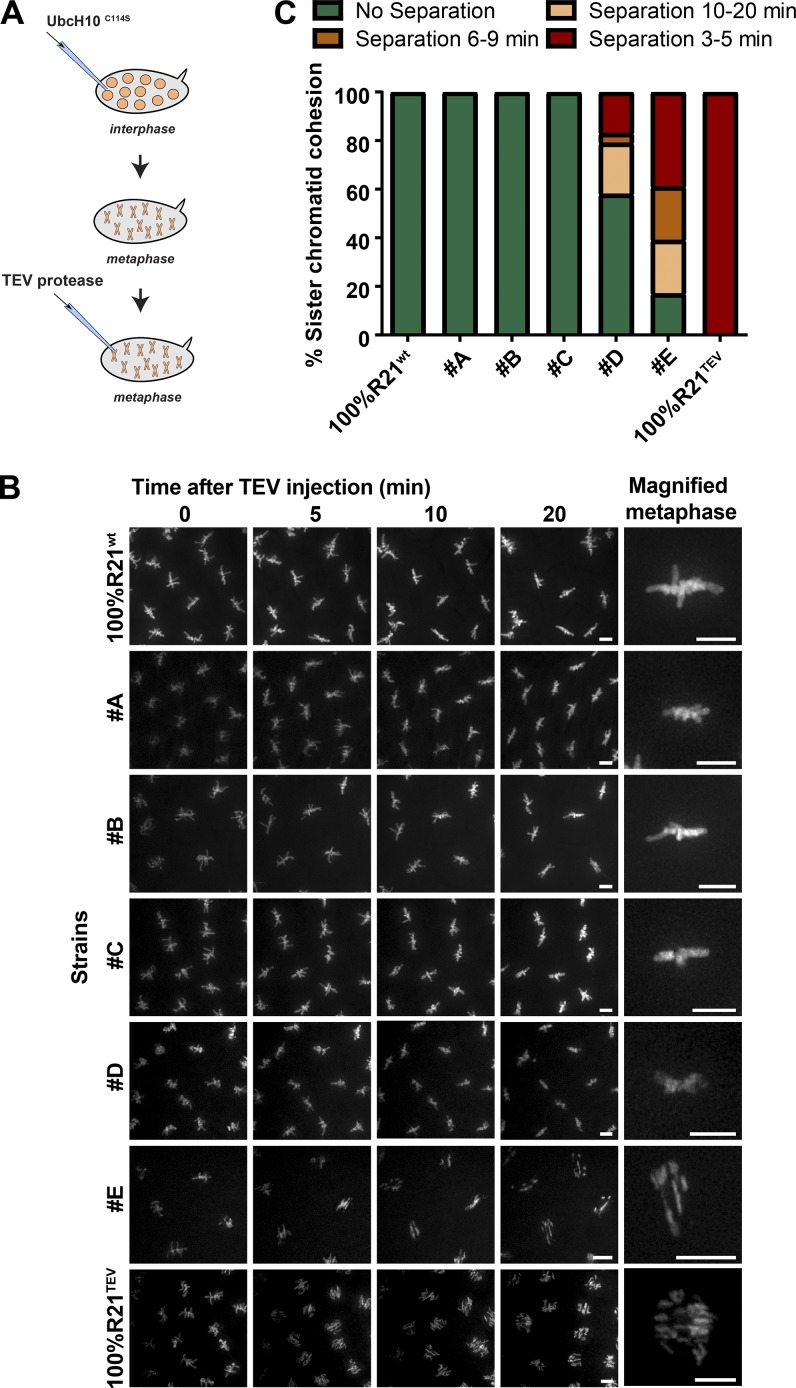
**Sister chromatid cohesion is highly resistant to cohesin loss. (A)** Experimental layout: embryos carrying different ratios of TEV-cleavable/TEV-resistant complexes were arrested in metaphase using UbcH10^C114S^ and subsequently injected with TEV protease to trigger acute removal of specific cohesin percentages. **(B)** Metaphase-arrested chromosome behavior (labeled with H2B-mRFP) monitored for 20 min after TEV protease injection in the developed strains. The last column presents a magnified view of a metaphase 20 min after TEV injection. Bars, 5 µm. **(C)** Sister chromatid separation in the various strains at different times after TEV injection. *n* = 23, 5, 11, 9, 19, 23, and 13 embryos, respectively.

To estimate minimal cohesin amount necessary to sustain sister chromatid cohesion, we used the strains described above and induced acute loss of specific cohesin quantities. Embryos were arrested in metaphase using a dominant-negative form of the E2 ubiquitin ligase UbcH10^C114S^ (Fig. 2 A; Rape et al., 2006; Oliveira et al., 2010). This arrest preserves mitotic spindle integrity, and thus chromosomes are under constant pulling forces. However, under these conditions, no cohesion fatigue could be observed for the time course of the experiments (20 min) in chromosomes containing the full complement of cohesin (Fig. 2, B and C). Metaphase-arrested chromosomes containing variable amounts of TEV-resistant/TEV-sensitive complexes were subsequently injected with TEV protease to acutely release specific cohesin amounts from chromosomes. We observed that removal of >50% of cohesin caused no detectable change in the cohesion state within the time frame of the experiments. Full sister chromatid separation upon TEV addition could only be consistently detected in the strain E. This strain survives solely on 14% Rad21^WT^ (based on Rad21^TEV^-myc quantifications; Fig. S1, A and D) and presents 17% of TEV-resistant cohesin complexes on mitotic chromosomes as estimated by live imaging (Fig. 1 D).

Cohesin removal to levels above this threshold (strain D), with ∼22% of cohesin complexes persisting on mitotic chromosomes (based on live-imaging analysis; Fig. 1 D), resulted in an intermediate phenotype. In a small subset of analyzed embryos, partial or full chromosome separation was detected within a 20-min period (Fig. 2 C). However, in most embryos from this strain, chromosomes remained cohered for the entire duration of the experiment. Occasionally, we observed that chromosome 4, the smallest chromosome in the fly, detached from metaphases. Due to its reduced size, this chromosome has lower cohesin levels and is thus more prone to disjunction upon cohesin loss (not depicted). These results further emphasize that the remaining cohesin complexes present in this strain are close to the minimal threshold to sustain sister chromatid cohesion. Thus, sister chromatid cohesion is quite resistant to cohesin levels, and removal of 78% of cohesive links (or 95% in light of the handcuff model; see Fig. 1 B, dashed lines) sufficed sister chromatid cohesion in the majority of analyzed embryos.

### Partial cohesin loss compromises kinetochore–microtubule attachments

**Figure 3.**
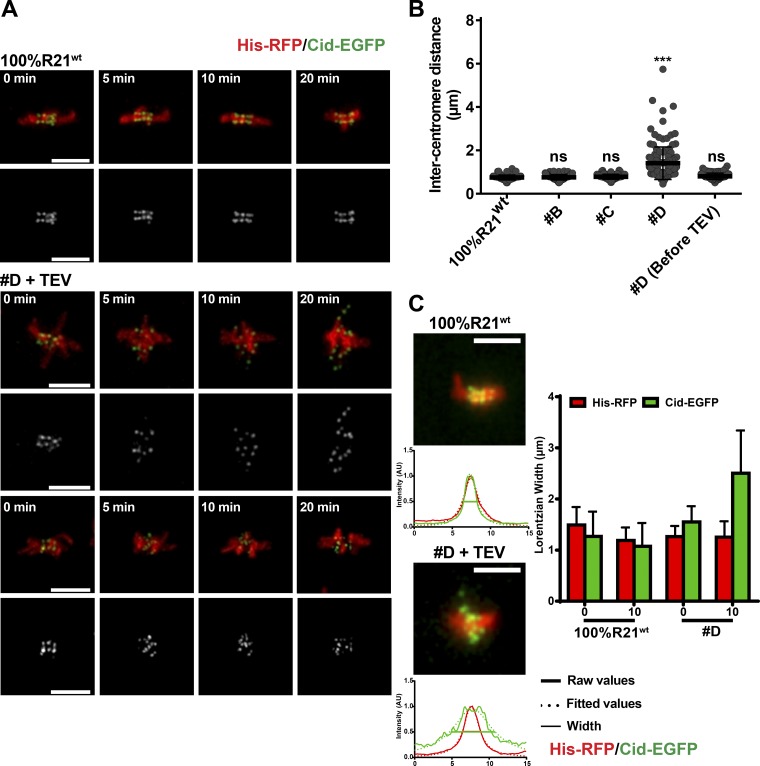
**Cohesin decay leads to abnormal centromere alignment. (A)** Representative images of centromere positioning (Cid-EGFP, green) in control (100% Rad21^WT^) and D strains. DNA is labeled with His-RFP (red). Times are relative to TEV injection. **(B)** Intercentromere distances in metaphase upon Rad21^TEV^ cleavage 10 min after TEV injection and before TEV addition for strain D. 20 centromere pairs were analyzed per embryo (*n* = 6, 5, 6, 7, and 6 embryos). ***, P < 0.0001, Kruskal-Wallis test relative to 100% Rad21^WT^. **(C)** Chromosome/centromere alignment of His-RFP and Cid-EGFP profiles at 0 and 10 min after TEV injection. His-RFP/Cid-EGFP intensity plot profiles were fit to a Lorentzian function as illustrated, and the width value was used as an alignment readout. Five metaphases were measured per embryo (*n* = 7 and 6 embryos for control and strain D, respectively). For these analyses, only embryos that did not display chromatid disjunction within the course of the experiment (20 min) were analyzed. Bars, 5 µm.

The results above indicate that in a significant fraction of analyzed embryos, chromosomes depleted of ∼80% of cohesin complexes are still able to sustain cohesion without individualization of sister chromatids. To further characterize the effect of such partial cohesion loss, we analyzed the behavior of centromeres in this strain upon sudden removal of the cleavable cohesin fraction. We focused this analysis in embryos that do not show evident sister chromatid disjunction of the main chromosomes within the time frame of the experiments (20 min). In contrast with the cohered major chromosome mass, analysis of centromeres revealed a very different behavior. First, we observed a significant increase in centromere separation 10 min after TEV injection (Fig. 3, A and B). Second, chromosome alignment was severely compromised, whereas positioning of the major chromosome mass remained unchanged (Fig. 3, A and C; and Fig. S2 A). This was caused mostly by changes within the inner centromere as revealed by significant separation of pericentromeric chromatin domains evaluated by transcription activator-like effector (TALE)-lights specific to the 1.686 repeat (Fig. S2, B and C; Yuan et al., 2014). In contrast, outer kinetochore structure seemed unchanged upon partial cohesin loss (Fig. S2 D). Lastly, chromosome misalignment was accompanied by highly dynamic movements of centromeres, which engaged into oscillations across the metaphase plate (Video 1).

**Figure 4.**
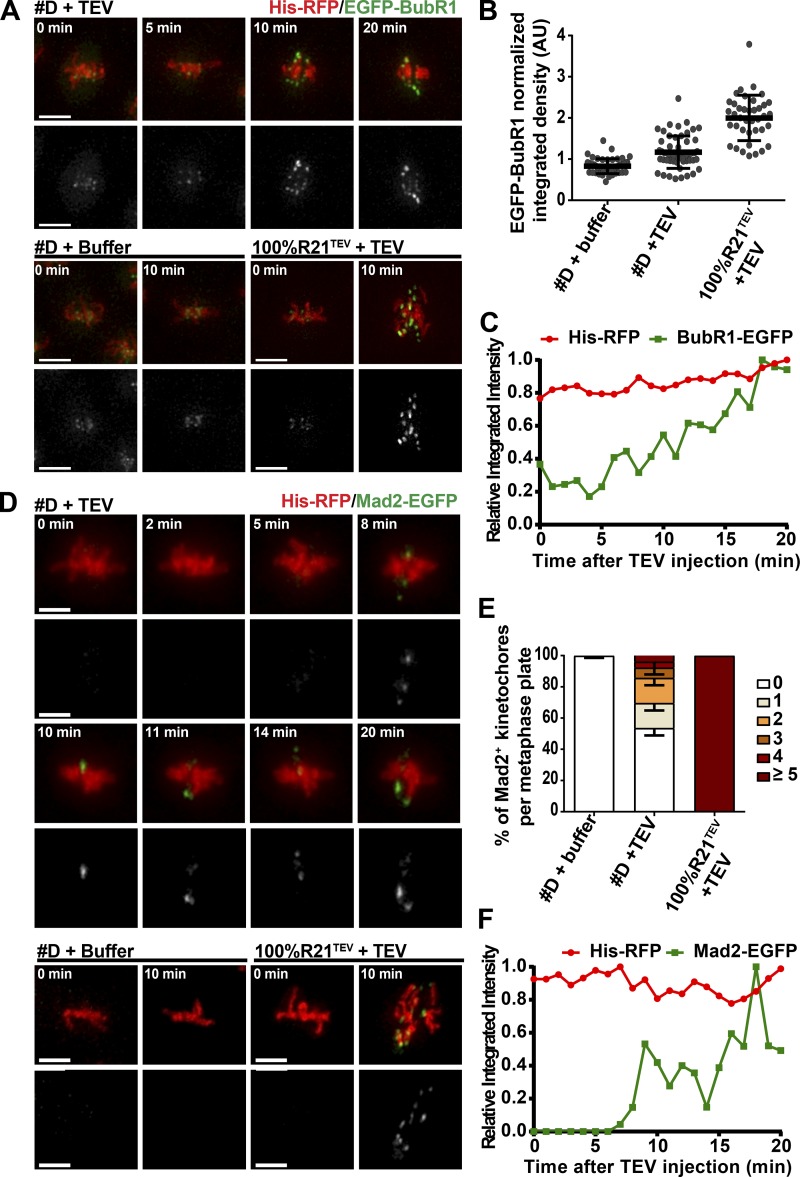
**Cohesin decay destabilizes kinetochore–microtubule interactions. (A)** EGFP-BubR1 localization on chromosomes in strain D + TEV (top), strain D + TEV buffer (bottom left, negative control), and strain containing 100% Rad21^TEV^ + TEV (bottom right, positive control). Times are relative to TEV injection. **(B)** Relative integrated intensity of EGFP-BubR1 fluorescence in the indicated strains normalized to time of TEV injection (*t*_10_/*t*_0_). *n* = 40/4; *n* = 56/6; and *n* = 39/4 (*n*, number of metaphases analyzed/number of independent embryos). **(C)** Profile of EGFP-BubR1 levels from a single metaphase of strain D + TEV across time. **(D)** Mad2-EGFP localization in strain D + TEV (top), D + TEV buffer (bottom left, negative control), and strain containing 100% Rad21^TEV^ + TEV (bottom right, positive control). Times are relative to TEV injection. **(E)** Frequency distribution of metaphase plates presenting different numbers of Mad2-EGFP–positive signals per metaphase 10 min after TEV protease or TEV buffer injection. *n* = 4, 8, and 5 embryos (>100 metaphases were analyzed per condition). **(F)** Profile of Mad2-EGFP levels from a single metaphase of strain D + TEV across time. For all measurements, only embryos that did not display chromatid disjunction within the course of the experiment (20 min) were analyzed. Mean ± SD. Bars, 5 µm. See also Fig. S2.

Although lower in amplitude, this oscillatory behavior resembled the dynamic motion of isolated chromatids upon full sister chromatid separation, which undergo cycles of attachment and detachment driven by the error-correction machinery (Oliveira et al., 2010; Mirkovic et al., 2015). We thus hypothesized that chromosome alignment defects and centromere oscillatory movements could stem from erroneous detachments upon sudden loss of most cohesive links. Much research supports that error-correction kinase Aurora B is able to sense the amount of tension at kinetochores and destabilizes tensionless attachments (Biggins, 2015). Acute removal of most cohesin links did not change Aurora B levels at the inner centromere (Fig. S2, E and F). We therefore first estimated whether partial cohesin loss could change the amount of tension sensed at kinetochores. We probed for levels of the spindle assembly checkpoint (SAC) component BubR1, known to label kinetochores that lack tension (Logarinho et al., 2004). Upon removal of ∼80% of cohesin complexes, kinetochores from embryos that did not disjoin within 20 min displayed a significant increase in BubR1 amount already 10 min after TEV injection. Levels increased gradually and reached on average ∼30% of the levels observed upon full cohesin loss (Fig. 4, A–C).

Next, we monitored the state of chromosome attachment in this experimental condition. We showed that upon sudden cohesin removal, compromised inner centromere structure is often associated with loss of kinetochore–microtubule attachments as judged by the occasional appearance of the SAC protein Mad2-EGFP at kinetochores (Fig. 4, D and E). Time-course analysis reveals that upon sudden loss of large cohesin amounts, cohered chromatids transiently appear labeled with Mad2-EGFP signal, although the intensity and positioning of the signals oscillates rather than presenting a steady increase (Fig. 4 F). These findings suggest attachments are constantly established and released as expected from error correction reactions. We therefore conclude that upon removal of a large fraction of cohesin complexes, remainder amounts are still sufficient to sustain sister chromatid cohesion in most embryos but not the integrity of the inner-centromere region, impairing maintenance of chromosome attachments and alignment.

### Partial cohesin loss impairs mitotic fidelity

**Figure 5.**
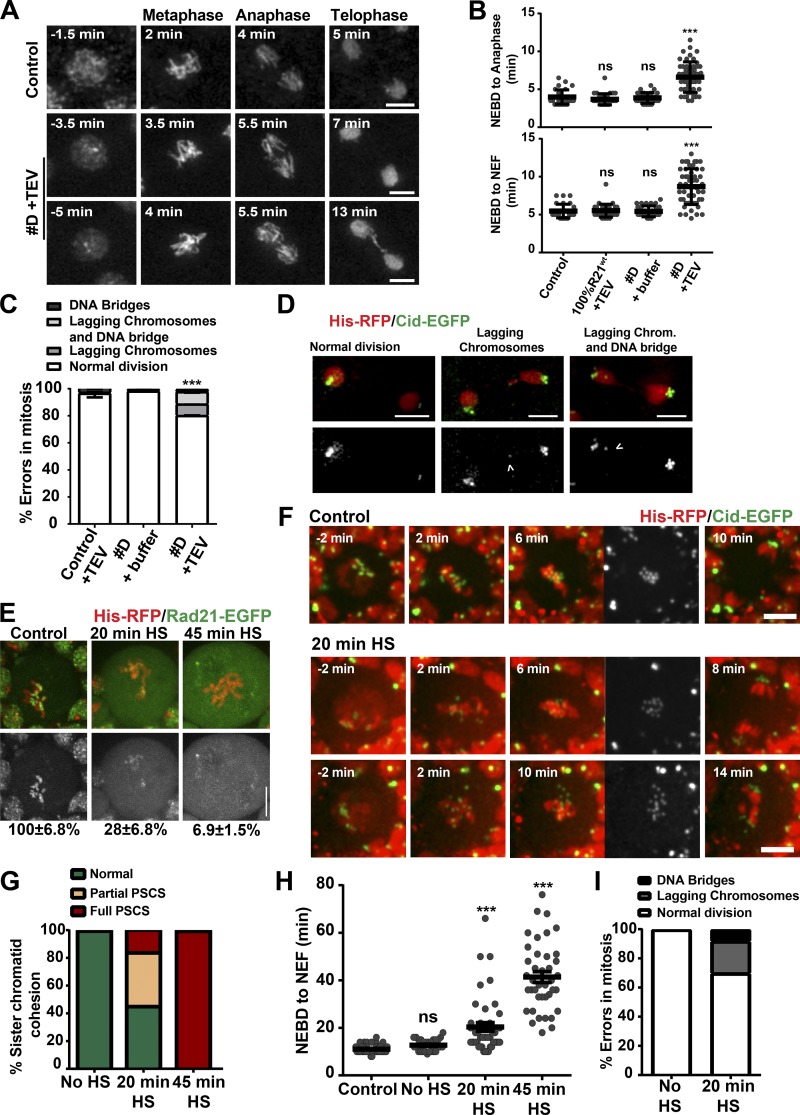
**Partial cohesin cleavage impairs mitotic progression. (A)** Stills of embryos undergoing mitosis with full complement of cohesin complexes (100% Rad21^WT^, control) and upon removal of a large percentage of cohesin from chromosomes before mitotic entry (strain D after TEV injection). The first column depicts time of injection. Times are relative to NEBD. **(B)** Mitotic timings (NEBD to anaphase [top] and NEBD to nuclear envelope formation [NEF; bottom]) in different conditions. Five nuclei per embryo were followed across time. *n* = 5 (control, genetic WT Rad21), 6 (100% Rad21^WT^), 4 (strain D + TEV buffer), and 10 (strain D + TEV protease) embryos. ***, P < 0.001, Kruskal-Wallis test relative to control. **(C)** Mitotic errors observed during chromosome segregation in strain D upon Rad21^TEV^ cleavage. Mitotic errors were measured in 5 (control), 5 (strain D + buffer), and 7 (strain D + TEV protease) embryos corresponding with at least 111 mitotic figures analyzed per condition. Mean ± SD. **(D)** Representative images of common errors observed in strain D highlighting lagging Cid-EGFP signals (arrowheads). Normal division comes from a control condition. **(E)** Native chromosome squashes from brains in no HS control and 20/45 min after HS-induced TEV expression in strains carrying solely Rad21^TEV^-EGFP (green). Percentages depict average levels of chromatin-bound Rad21^TEV^-EGFP (see also Fig. S3 A). Mean ± SEM. Cells also express His-H2Av-mRFP1 (red). **(F)** Stills from neuroblasts in control and mild cohesin cleavage conditions (20 min HS). Partial cleavage of cohesin results in mild to severe metaphase centromere misalignment and mitotic errors. Times are relative to NEBD. Bars, 5 µm. **(G)** Frequency of partial/full PSCS in the indicated conditions. **(H)** Mitotic timings across different experimental conditions: control strains surviving on Rad21^TEV^ without HS, cells containing WT Rad21 (TEV resistant) after HS, and cells surviving solely on Rad21^TEV^ after 20 or 45 min induction of TEV protease. *n* ≥ 4 independent brains corresponding with ≥40 cells analyzed per experimental condition. **(I)** Frequency of segregation errors observed upon partial cohesin cleavage (20 min HS). Cells undergoing full PSCS were excluded from analysis. *n* = 5 brains; *n* = 27 cells.

To evaluate the effect of partial cohesion decay on mitotic fidelity, we tested how embryos from strain D would divide upon removal of most cohesins at mitotic entry. Embryos were injected when mitotic chromosome compaction was already evident, and therefore, replication (and hence cohesion establishment) completed. Embryos that displayed full sister chromatid disjunction were excluded from subsequent analysis to focus solely in embryos where chromatid conjunction is not impaired. TEV-induced inactivation of a large subset of cohesin complexes led to a slight increase in mitotic duration (Fig. 5, A and B). Moreover, these experiments also resulted in a significant frequency of mitotic errors including chromosome lagging and anaphase bridges (Fig. 5, C and D; and Video 2).

Our results highlight that in *Drosophila* syncytial embryos, chromosomes are highly resistant to cohesin loss and that partial cohesin decay leads to compromised inner-centromere organization. *Drosophila* early embryos are known to undergo mitosis with high levels of maternally deposited proteins. It is therefore conceivable that increased cohesin levels could account for the resistance to cohesion decay. To investigate whether similar behavior would be observed in other cell types, we tested the effect of partial cohesin decay in cells where protein levels are dependent on cell-autonomous expression using larval brain neuroblasts as a model system. Our previous research reported that TEV-mediated cleavage of cohesin results in efficient loss of sister chromatid cohesion following heat shock (HS)-inducible TEV expression (Mirkovic et al., 2015). In agreement, quantitative analysis of chromosome spreads revealed that under these conditions, chromosome-bound cohesin levels were close to cytosolic amounts, further supporting removal of most cohesin complexes (Figs. 5 E and S3 A). To produce partial cohesin decay, we reduced the time of HS and consequently lowered TEV protease expression (Fig. S3, B–D), thereby reducing chromosome-bound cohesin to levels ∼27% of the ones observed in WT cells (Figs. 5 E and S3 A). Whereas 45 min HS resulted in total loss of sister chromatid cohesion across all cells analyzed, reduction of HS duration to 20 min caused a more graded response. We observed significant phenotype variability across different cells even within the same developing brain. Although 45% displayed a normal mitosis, a small percentage of cells (16%) underwent full PSCS with evident signs of chromatid individualization either immediately after nuclear envelope breakdown (NEBD) or during the mitotic delay (Fig. 5, F and G). This mosaic effect further supports that this experimental layout results in cohesion decay close to the minimal threshold for sister chromatid cohesion maintenance. Importantly, the remainder cells (∼39%) did not display sister chromatid disjunction but exhibited an abnormal metaphase organization, often with increased inter-centromere distances and chromosome misalignment (Figs. 5 F and S3 E). Despite the low frequency of full sister chromatid separation, cells underwent mitosis with a significant delay (Fig. 5 H). Mitotic errors are sometimes observed during mitotic exit, including lagging chromosomes and chromatin bridges (Fig. 5 I). We thus conclude that a large decay in cohesin levels impairs centromere rigidity necessary for efficient chromosome alignment and mitotic fidelity, even in conditions where sister chromatid cohesion is maintained.

Previous research in budding yeast reported that cells surviving on 13% Rad21/Mcd1 lack sister chromatid cohesion defects (Heidinger-Pauli et al., 2010). In agreement, our quantitative analysis reveals full sister chromatid disjunction in a metazoan organism is also very resistant to cohesin loss. However, cohesion decay compromises mitotic fidelity even in conditions that suffice chromosomal cohesive state. If ∼20% of chromosome-bound cohesin is sufficient to sustain cohesion, why do mitotic chromosomes have such excess in cohesin levels? Cohesin overload could possibly work as a protection mechanism against cohesion fatigue. However, mitosis in *Drosophila* cells, particularly in syncytial embryos, occurs very rapidly, making this an unlikely scenario. Alternatively, increased amounts of cohesin may account for specific functions of this complex beyond sister chromatid cohesion. Novel functions for cohesin within the inner centromere are now emerging (Mirkovic and Oliveira, 2017). In this study, we propose that cohesin density is necessary to provide chromosomes with rigidity to ensure precise force balance with the mitotic spindle and thereby guarantee proper chromosome attachment and alignment. Force equalization across the mitotic spindle has been previously demonstrated to contribute to anaphase synchrony (Matos et al., 2009), which may thus account for mitotic defects observed upon partial cohesin loss.

The exact role of the inner centromere as an important force contributor required for the mechanics of mitosis has been extensively debated. Cohesin has been proposed to play a central role in generating dynamic tension between microtubules to enable chromosomal attachments (Tanaka et al., 2000). Biophysical studies in budding yeast further highlighted cohesin’s role as major regulator of an elastic chromatin spring, an integrated part of the mitotic apparatus (Bouck and Bloom, 2007; Stephens et al., 2011, 2013; Lawrimore et al., 2015). Recent studies, in contrast, argue that mechanical tension exerted within the kinetochore might be more important to stabilize attachments than interkinetochore stretch (Maresca and Salmon, 2009, 2010; Uchida et al., 2009; Nannas and Murray, 2014). Our results highlight the importance of inner-centromere mechanical properties in the maintenance of stable chromosome attachments/alignment even once metaphase alignment has occurred. In metazoans, this role has been mostly attributed to the condensin I complex (Oliveira et al., 2005; Gerlich et al., 2006; Ribeiro et al., 2009; Piskadlo et al., 2017). Whether cohesin and condensin work collaboratively or independently in maintaining inner-centromere structure remains unknown.

In human pathologies, cohesin loss has been reported to be rather mild. Aged human oocytes show a decrease of ∼24–38% for meiotic cohesin subunits when compared with younger women (Tsutsumi et al., 2014). In light of our research, such decay is unlikely to promote sister chromatid disjunction, and meiotic errors associated with cohesin loss may be instead related with chromosomal geometry. Accordingly, studies in human oocytes revealed an increased distance between bivalents in meiosis of older females, leading to aberrant kinetochore attachments and segregation errors (Patel et al., 2015; Zielinska et al., 2015). Cohesin deregulation has also been associated with rare developmental conditions known as cohesinopathies (Dorsett, 2007; Liu and Krantz, 2008; Remeseiro et al., 2013). The absence of obvious cohesion defects in models for these diseases led to the assumption that transcription deregulation rather than mitotic failure underlies disease development. However, mild cohesion defects have been reported for Roberts and Warsaw breakage syndromes (Tomkins et al., 1979; Jabs et al., 1991; van der Lelij et al., 2010; de Lange et al., 2015). Errors in centromere organization (and consequently on chromosome alignment and attachment) may underlie previously unnoticed and milder mitotic defects despite functional chromosome cohesion.

## Materials and methods

### Fly strains

A full list of *Drosophila* stocks used can be found in Table 1. Strains expressing Rad21^TEV^-myc and Rad21^WT^-EGFP were previously described (Pauli et al., 2008; Oliveira et al., 2014). Expression of TEV protease in brain neuroblasts was achieved using HS-inducible TEV protease by heat-shocking third instar larvae at 37°C for the specified time (Mirkovic et al., 2015). To prevent leaky TEV protease expression, larvae were grown at 18°C before HS. Upon HS, larvae were then left to recover at room temperature before processed for live-cell imaging or Western blotting. Fly strains also expressed His2Av-mRFP and Cid-EGFP (Schuh et al., 2007) as well as GFP-Mad2 and GFP-BubR1 (Buffin et al., 2005) fluorescent markers.

**Table 1.** List of fly strains used in this study

**Stock #**^a^	**Genotype**	**Reference**
269	*w;; polyubiq-H2B-RFP*	Oliveira et al. (2010)
477	*w; hspr-NLSv5TEV /CyO; Rad21^ex3^/TM6B_ubiGFP_*	Pauli et al. (2008)
629	*w;; rad21^ex15^, polyubiq-H2B-RFP, tubpr-Rad21(550-3TEV) -myc10 (4c)*	Oliveira et al. (2010)
820	*w;; HisH2AvD-mRFP1 III.1, CGC (CID-EGFP) III.1*	Schuh et al. (2007)
868	*If/CyO; Rad21^ex15^, tubpr-Rad21(550-3TEV) (4c), CGC III.1/(TM3,Ser)*	Oliveira et al. (2010)
1090	*w; GFP-BubRI/(CyO); rad21^ex15^, polyubiq-H2B-RFP, tubpr-Rad21(550-3TEV) -myc10 (4c)*	Mirkovic et al. (2015)
1150	*w;; le_1_-tubpr-Rad21(wt) –EGFP (7)/TM3, Ser*	This study
1224	*w;; rad21^ex15^, polyubiq-H2B-RFP, tubpr-Rad21(TEV) –EGFP (2)*	Oliveira et al. (2014)
1225	*w;; rad21^ex15^, polyubiq-H2B-RFP, tubpr-Rad21(wt) –EGFP (2)*	Oliveira et al. (2014)
1236	*w; P[w+, gCRC]II.1, P[w+, gCRC]II.2/CyO; tubprom-Rad21(wt)-EGFP 2, Rad21^ex15^*	Oliveira et al. (2014)
1580	*w; HisH2AvD mRFP1 II.2/CyO; rad21^ex15^,tubpr-Rad21(550-3TEV) -myc10 (4c), CGC III.1 /TM3,Ser*	Oliveira et al. (2010)
1679	*w-; GFP-Mad2; rad21^ex15^, poliubiq-His-RFP, tubpr-Rad21(550-3TEV) -myc10 (4c)*	Mirkovic et al. (2015)
1695	*w;; rad21^ex15^, polyubiq-H2B-RFP, tubpr-Rad21(550-3TEV) -myc10 (4c), tubpr-Rad21(wt) –EGFP (2)*	This study
1704	*w;; rad21^ex15^, polyubiq-H2B-RFP, le_1_-tubpr-Rad21(wt) –EGFP (7) /TM6B*	This study
1705	*w;; rad21^ex15^, le_1_-tubpr-Rad21(wt) –EGFP (7), tubpr-Rad21(550-3TEV) -myc10 (4c)*	This study
1745	*w; P[w+, gCRC]II.1, P[w+, gCRC]II.2/CyO; rad21^ex15^, polyubiq-H2B-RFP, le_1_-tubpr-Rad21(wt) –EGFP (7)/TM6B,Hu*	This study
1746	*w; p[w+,gSpc105-mRFP]II.1 /CyO; rad21^ex15^, polyubiq-H2B-RFP, le1-tubpr-Rad21(wt) –EGFP (7)/TM6B,Hu*	This study

a Reference number in our internal laboratory fly database.

### Microinjections

Microinjection experiments were performed as previously described (Oliveira et al., 2010; Piskadlo et al., 2017). Dechorionated embryos (1–1.5 h old) were glued to a #1.5 coverslip and covered with Series 700 halocarbon oil (H8898; Sigma-Aldrich). Embryos were then injected at 18–20°C into the posterior pole using a Burleigh Thorlabs micromanipulator, a Femtojet microinjection system (Eppendorf), and prepulled Femtotip I needles (Eppendorf). Injections were performed using 12 mg/ml UbcH10^C114S^ diluted in 20 mM Tris-HCl, pH 7.5, 300 mM NaCl, 6 mg/ml TEV protease in TEV buffer (20 mM Tris-HCl, pH 8.0, 1 mM EDTA, 50 mM NaCl, and 2 mM DTT), or 2 mM colchicine diluted in PBS, pH 7.4. TALE-light GFP 1.686 was produced as previously described (Yuan et al., 2014) and was injected at 1 mg/ml in 40 mM Hepes and 150 mM KCl, pH 7.4. Aurora B–EGFP mRNA preparation/injection was performed as previously described (Oliveira et al., 2010) and used at a concentration of 74 ng/µl.

### Immunoblotting analysis

Staged embryos (10–14 cycles) were selected using a stereo zoom microscope and collected according to the procedure by Prudêncio and Guilgur (2015). Adult female ovaries from adult females samples were collected and mechanically disrupted in radioimmunoprecipitation assay buffer. Extracts were cleared by a prespin at 20,000 *g* for 5 min at 4°C after water bath sonication (Power 5 Sonicator XL2020; Misonix). Brain samples were prepared by homogenization of dissected brains in loading buffer. Samples were loaded on a 10%/13% SDS gel for electrophoresis and then were transferred to nitrocellulose membranes. Western blot analysis was performed according to standard protocols using the following antibodies: anti–myc tag (1:200; sc-47694; RRID: AB_627266; Santa Cruz Biotechnology, Inc.), anti–α-tubulin (1:50,000; DM1A; T9026; RRID: AB_477593; Sigma-Aldrich), anti-Rad21 (1:5,000; Heidmann et al., 2004), anti-V5 (1:300; ab9116; RRID: AB_307024; Abcam), and anti-lamin (1:1,000, deposited to the Developmental Studies Hybridoma Bank by P.A. Fisher, State University of New York at Stony Brook, Stony Brook, NY; adl84.12; RRID: AB_528338). Antibodies were detected with HRP-conjugated secondary antibodies (Jackson ImmunoResearch Laboratories, Inc.) and developed with Pierce ECL Western blotting substrate (Thermo Fisher Scientific) or infrared-conjugated secondary antibodies (LI-COR Biosciences) and visualized on a LI-COR Odyssey (LI-COR Biosciences) according to the manufacturer’s instructions. For quantitative Western blot analysis of total Rad21 and myc-tagged Rad21^TEV^ levels in the different strains, protein levels were estimated using a titration curve containing variable of embryos of the *w-;;rad21^ex^,Rad21^TEV^-myc* strain (100% R21^TEV^) run concomitantly with the test samples. All quantifications were performed using FIJI software (RRID: SCR_002285; National Institutes of Health; Schindelin et al., 2012).

### In vitro cleavage experiments

Ovaries were dissected as described above but using PBS, pH 7.4. The recovered supernatant and protein concentration was determined using Bradford assays. For cleavage experiments, 20 µg soluble extract was incubated with 1 µg TEV protease during the described incubation periods (5, 10, 20, and 120 min).

### Embryo chromosome spreads

Analysis of Rad21^WT^-EGFP levels was performed in embryos following a previously described protocol with minor modifications (Schittenhelm et al., 2007). Briefly, collections of 75-min-old embryos were dechorionated and incubated in a 1:1 PBS, pH 7.4, and 1 mM colchicine and heptane solution for 15 min with agitation. Embryos were then washed once in PBS and 1 mM colchicine. 10–20 embryos were transferred to a 6–8-µl drop of PBS, 100 µM colchicine, and 2 µg/ml Hoechst 33258 on a 22 × 40–mm coverslip. Embryos were squashed by capillary forces after laying a 22 × 22–mm coverslip. Images were acquired up to 30 min after chromosome spreads.

### Brain chromosome spreads

Analysis of Rad21^TEV^-EGFP levels in neuroblast chromosomes was performed as previously described (Oliveira et al., 2014) with minor modifications. Briefly, larval brains were incubated with 100 µM colchicine in Schneider’s medium for 45 min. Afterward, brains were placed in a 6-µl drop of a PBS, 100 µM colchicine, and 2 µg/ml Hoechst 33258 solution and then squashed between two coverslips. Images were taken up to 30 min after tissue sample preparation.

### Live-cell imaging

Live-cell imaging of larval neuroblasts and analysis of Rad21^WT^-EGFP levels in embryo chromosomal spreads was performed on a spinning-disk confocal using a Revolution XD microscope (Andor Technology) equipped with a 60× glycerol immersion 1.30 NA objective (Leica Microsystems) or a 100× 1.40 NA oil objective (Leica Microsystems) and an iXon Ultra 888 1,024 × 1,024 electron-multiplying charge-coupled device (Andor Technology). All the remaining live imaging of *Drosophila* embryos was performed on an inverted widefield DeltaVision microscope (Applied Precision Ltd.) at 18–20°C in a temperature-controlled room using a 100× oil-immersion 1.4 NA objective lens (Olympus), an electron-multiplying charge-coupled device camera (Roper Cascade 1024), and standard live filter sets. 3D images were acquired every minute with z series optical sections recorded every 0.8 µm with SoftWoRx software (5.5.0; Applied Precision Ltd.). Widefield images were restored by conservative deconvolution with SoftWoRx software. Images were assembled using FIJI software (Schindelin et al., 2012), and selected stills were processed with Photoshop CS6 (Adobe), FIJI, or OMERO.figure (Allan et al., 2012).

### Quantitative image analysis

Rad21^WT^-EGFP (embryos) and Rad21^TEV^-EGFP (neuroblasts) levels on chromosomes were accessed on the z projections of the images using DNA staining as a mask. Images were analyzed using FIJI software (Schindelin et al., 2012). Disjunction of sister chromatids, Mad2-positive signal at metaphase plate, mitotic timings, and errors in mitosis were estimated manually using FIJI. To measure intercentromere distances and distances between 1.686 repeat EGFP–TALE-light, a 3-px-wide line was placed over sister (peri)centromere pairs, and the distance was measured by the length between the corresponding peaks on a Cid-EGFP or TALE-light plot profile. Chromosome/centromere alignment was measured by placing a 40-px-wide line along the segregation plane, and plot profiles for His-RFP/Cid-EGFP were obtained using FIJI and normalized to the maximum intensity within each dataset. Values were then fitted to a Lorentzian function using Prism 7 (GraphPad Software), and the corresponding width was used as an alignment estimation. Mad2-EGFP–/EGFP-BubR1–/Aurora B–EGFP–integrated intensities were measured over time (after image threshold) and normalized to the first frame after TEV injection (time 0).

### Statistical analysis

Statistical analysis and graphs were performed using Prism 7. Data were tested for normality using the D'Agostino and Pearson normality test. Comparative analysis between groups was performed using the nonparametric Kruskal-Wallis test (Dunn’s multiple comparison test) or unpaired two-tailed Student’s *t* test. Sample size, error bars (SEM or SD), and p-values are reported on each figure.

### Online supplemental material

Fig. S1 (A–E) shows additional details on the developed strains including detailed genotypes, total levels of Rad21 in each strain, relative levels of Rad21^TEV^/Rad21^WT^ measured by Western blotting, and plot profiles with Rad21 localization along chromosome length. Fig. S1 F depicts the kinetics of in vitro cleavage of Rad21^TEV^, and Fig. S1 (G and H) shows an example of in vitro cleavage leading to partial cohesin inactivation. Fig. S2 shows alignment measurements for additional strains analyzed and additional analysis for analysis strain D relative to controls including pericentromere domains (EGFP–TALE-light for the 1.686 repeat), localization of outer-kinetochore proteins (Spc105), and levels of Aurora B at the inner centromere. Fig. S3 shows quantitative analysis of Rad21^TEV^-EGFP cleavage in the neuroblasts experiments. Video 1 depicts chromosome and centromere behavior upon TEV addition in strain D compared with controls, whereas Video 2 shows mitosis upon cleavage of ∼80% of cohesin complexes before mitotic entry (strain D + TEV).

## Supplementary Material

Supplemental Materials (PDF)

Video 1

Video 2
