# Supplementary material for: A quantitative analysis of cohesin decay in mitotic fidelity
Source: J Cell Biol. 2018 Oct 1;217(10):3343–53. doi: 10.1083/jcb.201801111 (PMC6168270; doi:10.1083/jcb.201801111)
Supplement: Supplemental Materials (PDF) [file JCB_201801111_sm.pdf]

Supplemental material

Carvalhal et al., https://doi.org/10.1083/jcb.201801111

A

Strain code	Genotype	Transgene copies Rad21wt-EGFP: Rad21TEV-myc: Rad21wt-EGFP (le)	% WT (predicted from genetics)[a]	Total Rad21[b]	%WT x Total levels	% WT (measured by w.b.)[c]	% WT (measured by l.img.)[d]
100%R21wt	*w-;;rad21ex, Rad21wt-EGFP*	2:0:0	100	108	108	100	100
#A	*w-;;rad21ex, Rad21wt-EGFP, Rad21TEV-myc*	2:2:0	52	179	93	59	85
#B	*w-;;rad21ex, Rad21wt-EGFP/ rad21ex, Rad21TEV-myc*	1:1:0	52	136	71	56	68
#C	*w-;;rad21ex, Rad21wt-EGFP, Rad21TEV-myc/ rad21ex, Rad21TEV-myc*	1:2:0	35	113	40	45	49
#D	*w-;;rad21ex, Rad21wt-EGFP (le)/ rad21ex, Rad21TEV-myc*	0:1:1	35	62	22	19	22
#E	*w-;;rad21ex, Rad21TEV-myc, Rad21wt-EGFP(le)/ rad21ex, Rad21TEV-myc*	0:2:1	21	105	22	14	17
100%R21TEV	*w-;; rad21ex, Rad21TEV-myc*	0:2:0	0	100	0	0	0

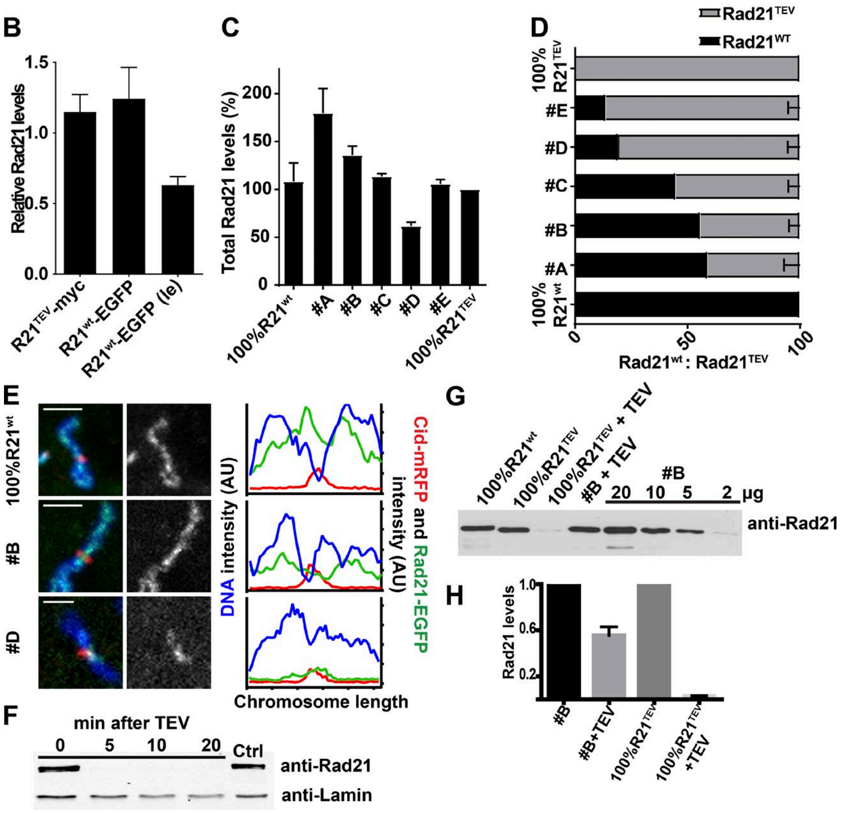

Figure S1. **Overview of the strains used in this study. (A)** Summary of the genetic combinations used in this study indicating specific genotypes and the corresponding ratios of each transgene (Rad21^{WT}-EGFP: Rad21^{TEV}-myc: Rad21^{wt}-EGFP [low expression; le]) as well as estimations of relative Rad21 amounts. a, amounts of Rad21^{WT} predicted from genetics were calculated based on the relative amounts of the expression of each transgene (see B); b, total levels of Rad21 (Rad21^{WT} + Rad21^{TEV}) based on the total amount of Rad21 expression measured in each strain relative to *w-;;rad21^{ex},Rad21^{TEV}-myc* (see C); c, relative levels of Rad21^{WT}-EGFP estimated by Western blot (w.b.) analysis (total Rad21-Rad21^{TEV}-myc as in D); d, relative levels of Rad21^{WT} measured by quantitative live-cell imaging (l.img.) normalized to the fluorescence intensity of *w-;;rad21^{ex},Rad21^{wt}-EGFP* (as in Fig. 1 D). **(B)** Quantification of Rad21 levels expressed by each transgene relative to WT levels (endogenous copy of Rad21 in WT strains). $n \geq 8$ independent experiments for each experimental condition. **(C)** Quantification of Rad21 levels in the different strains used in this study normalized to 100% R21^{TEV} (*w-;;rad21^{ex},Rad21^{TEV}-myc*). $n \geq 3$ independent experiments for each experimental condition. **(D)** Ratio between Rad21^{WT} and Rad21^{TEV}. Ratios were calculated based on the difference between total levels of Rad21 (C) and measured levels of Rad21^{TEV} (obtained by Western blot analysis against myc antibody that detects only myc-tagged Rad21^{TEV}). $n \geq 4$ independent experiments for each experimental condition. **(E)** Representative intensity plot profiles for DNA, Cid-mRFP, and Rad21^{WT}-EGFP along an isolated chromosome of strain 100% R21^{WT}, strain B, and strain D. Bar, 2 µm. **(F)** Western blot of the in vitro Rad21^{TEV} cleavage kinetics for the experimental timings used in this study. Protein extracts were prepared from adult female ovaries, incubated with TEV protease for the referred times, loaded at 20 µg per lane, and probed with a Rad21 antibody. Lamin was used as a loading control. **(G)** Western blot showing in vitro cleavage of Rad21^{TEV} in strains surviving solely on TEV-cleavable Rad21 (100% R21^{TEV}) and in the strain B (predicted genetic values contains 52% Rad21^{TEV} molecules). Protein extracts were prepared from adult female ovaries and loaded at 20 µg per lane for the cleavage experiments and decreased quantities for the titration curve. Rad21 antibody was used to estimate the amount of uncleaved Rad21. **(H)** Quantification of the in vitro cleavage experiments as illustrated in G. $n = 6$ (strain B) and 3 (100% Rad21^{TEV}). All graphs show mean ± SEM.

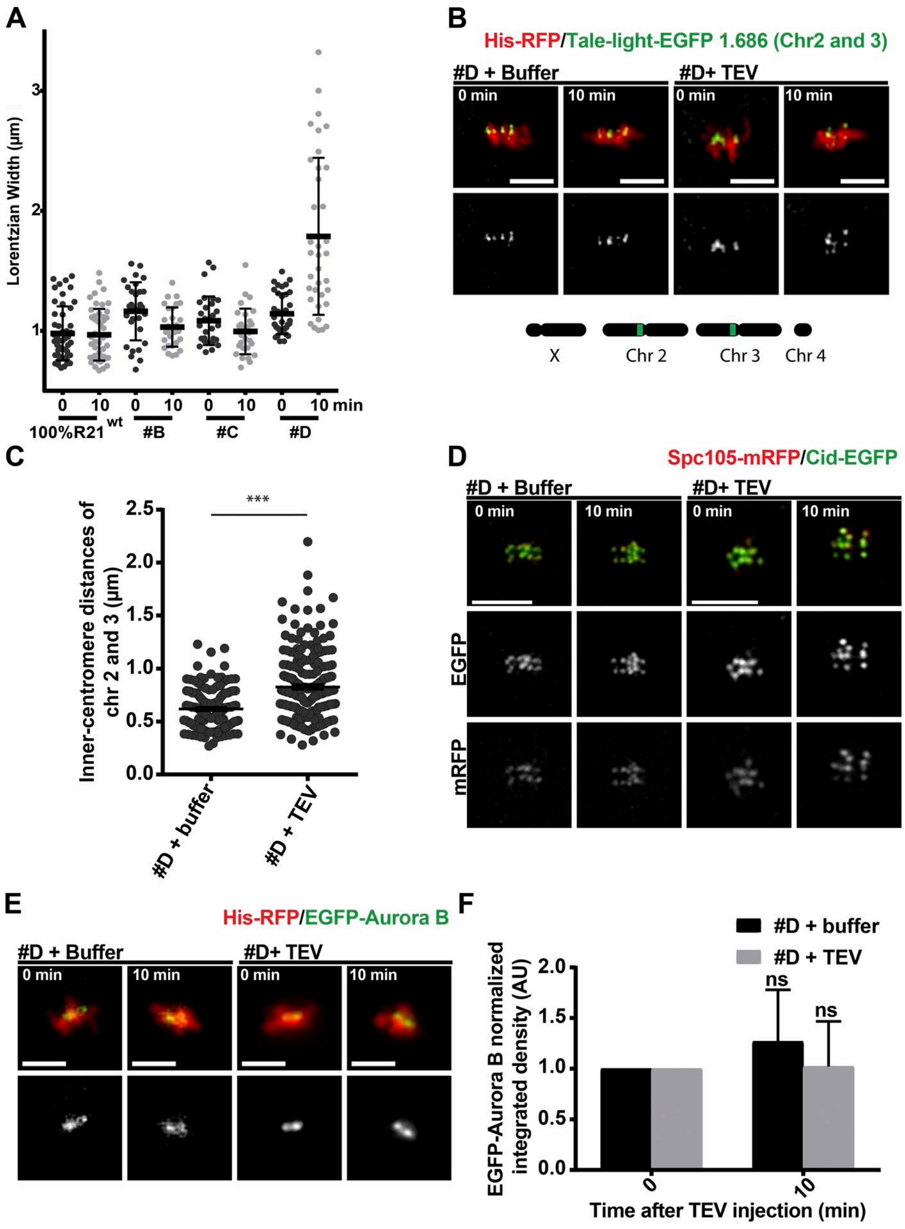

Figure S2. **Strain D preserves centromere and kinetochore organization upon TEV cleavage. (A)** Lorentzian fitted width obtained 0 and 10 min after TEV protease injection across the different strains (five metaphases were analyzed in 8, 6, 5, and 5 embryos, respectively). Strain D shows a wider dispersion of alignment when compared with the remained strains 10 min after TEV injection. **(B)** Representative localization of the 1.686 repeat EGFP–TALE-lights (labels pericentromeric chromatin on chromosomes 2 and 3) in strain D upon TEV cleavage. TEV buffer injection was used as control. **(C)** Quantitative analysis of the resolution of the 1.686 repeats (labeled with TALE-lights) measured 10 min after TEV buffer/TEV protease injection. n = 150, 4, and 209 metaphases, and n = 6 embryos, respectively). **(D)** Stills from a video showing Spc105-mRFP1 (red) and Cid-EGFP (green) in strain D embryos upon TEV buffer (control) or TEV protease injection. Spc105 does not change its localization across the time of the experiment (20 min). **(E)** Stills from a video showing EGFP–Aurora B signal in strain D embryos upon TEV buffer (control) or TEV protease injection. **(F)** Integrated intensity of EGFP–Aurora B of embryos from strain D injected with TEV buffer/TEV protease. t_{10} was normalized to t_0. n = 18/4 (buffer) and n = 27/6 (TEV; n, number of metaphases analyzed/number of independent embryos). Aurora B levels did not change across the time nor at the tested conditions (ns, ANOVA test). Only embryos that did not display chromatid disjunction within the course of the experiment (20 min) were used. Mean ± SD. Bars, 5 µm.

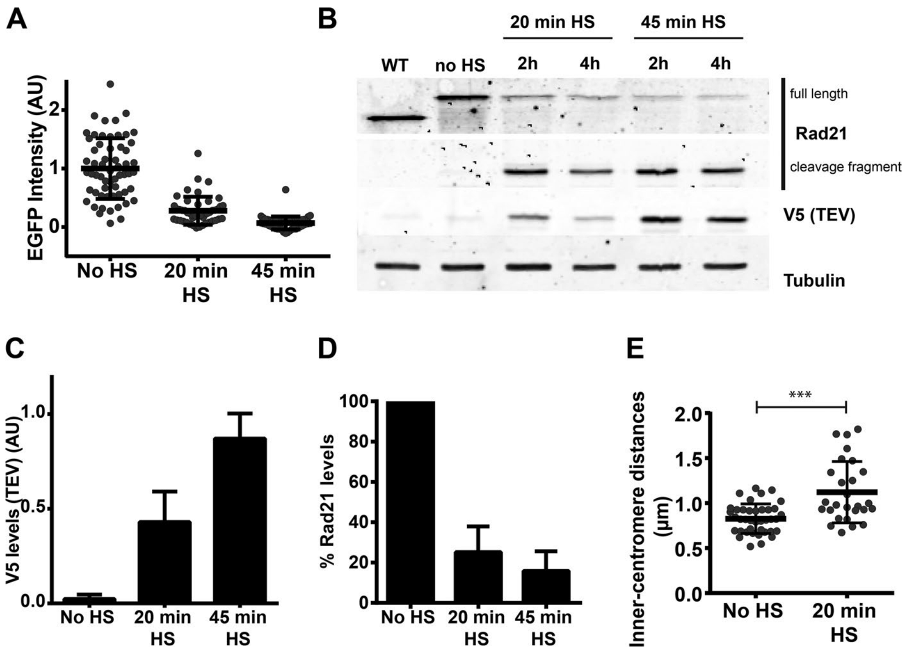

Figure S3. **Partial cohesin cleavage in larval neuroblasts. (A)** Quantifications of Rad21^{TEV}-EGFP levels within the chromosomal area after background subtraction normalized to the average values of the control strain (no HS). Quantifications were obtained from chromosome squashes as shown in Fig. 5 E. **(B)** Western blot analysis of larval brains at the indicated times before/after HS. **(C and D)** Quantitative analysis of HS-induced TEV protease expression (V5 tagged; C) or Rad21^{TEV}-EGFP (D) at the indicated times before/after HS. Averages were obtained from at least four independent samples prepared either before (no HS) or 2–4 h after HS. **(E)** Quantification of the inter-centromere distances in metaphase (2–4 min before anaphase onset) upon partial Rad21^{TEV} cleavage (20 min) compared with controls (no HS). n = 39 (no HS) and 27 (20 min HS) from at least 10 neuroblasts. ***, $P < 0.0001$, unpaired two-tailed t test.

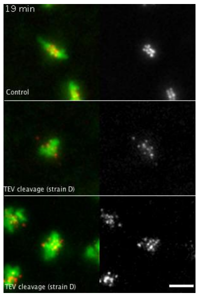

Video 1. **Partial loss of cohesin compromises centromere organization at metaphase plate.** Top: Chromosome dynamics of a representative metaphase (His-RFP [green] and Cid-EGFP [red]) from an unperturbed arrested embryo (UbcH10^{C114S} induced). Middle and bottom: Two representative metaphase plates from independent embryos of strain D after TEV cleavage. Times are relative to TEV injection (imaged every 1 min). Bar, 5 µm. Videos were obtained using widefield fluorescence microscopy with images acquired every 1 min (video shows four frames per second).

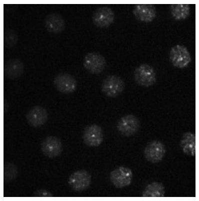

Video 2. **Partial cohesin loss impairs mitotic fidelity.** Chromosomes of a strain D embryo injected with TEV protease at the time of mitotic entry. Although most nuclei divide normally, mitotic errors are often observed during mitotic exit. Video was obtained using widefield fluorescence microscopy with images acquired every 30 s (video shows three frames per second).
